# Educational inequalities in diabetes mortality across Europe in the 2000s: the interaction with gender

**DOI:** 10.1007/s00038-015-0669-8

**Published:** 2015-03-07

**Authors:** Hadewijch Vandenheede, Patrick Deboosere, Albert Espelt, Matthias Bopp, Carme Borrell, Giuseppe Costa, Terje Andreas Eikemo, Roberto Gnavi, Rasmus Hoffmann, Ivana Kulhanova, Margarete Kulik, Mall Leinsalu, Pekka Martikainen, Gwenn Menvielle, Maica Rodriguez-Sanz, Jitka Rychtarikova, Johan P. Mackenbach

**Affiliations:** 1 Interface Demography, Department of Sociology, Vrije Universiteit Brussel, 5 Pleinlaan, 1050 Brussels, Belgium; 2 Department of Public Health, Erasmus Medical Centre, Erasmus University Rotterdam, 12 Wytemaweg, 3015 CN Rotterdam, The Netherlands; 3 Public Health Agency of Barcelona, Autonomous University of Barcelona, 1 Plaça de Lesseps, 08023 Barcelona, Spain; 4 Institute of Social and Preventive Medicine, University of Zurich, 84 Hirschengraben, 8001 Zurich, Switzerland; 5 Department of Public Health, University of Turin, 94 Piazza Polonia, 10126 Turin, Italy; 6 Department of Sociology and Political Science, Norwegian University of Science and Technology, Dragvoll, Building 9, Level 5, 7491 Trondheim, Norway; 7 Epidemiology Unit, Local Health Agency of Collegno and Pinerolo, 30 via Martiri XXX Aprile, 10093 Collegno, Turin Italy; 8 Present Address: Center for Tobacco Control Research and Education, University of California, San Francisco, 530 Parnassus Ave. Suite 366, San Francisco, CA 94143 USA; 9 Stockholm Centre on Health of Societies in Transition, Södertörn University, Moas båge, 5 Alfred Nobels Allé, 141 89 Huddinge, Sweden; 10 Department of Epidemiology and Biostatistics, National Institute for Health Development, 42 Hiiu, 11619 Tallinn, Estonia; 11 Department of Social Research, University of Helsinki, 37 Unioninkatu, 00014 Helsinki, Finland; 12 Department of Social Epidemiology, Pierre Louis Institute of Epidemiology and Public Health, French National Institute of Health and Medical Research, 101 Rue de Tolbiac, 75013 Paris, France; 13 Department of Social Epidemiology, Pierre Louis Institute of Epidemiology and Public Health, Sorbonne University, UPMC Univ Paris 06, Paris, France; 14 Department of Demography and Geodemography, Charles University in Prague, 6 Albertov, 128 43 Prague 2, Czech Republic

**Keywords:** Diabetes mellitus, Education, Europe, Gender, Inequalities

## Abstract

**Objectives:**

To evaluate educational inequalities in diabetes mortality in Europe in the 2000s, and to assess whether these inequalities differ between genders.

**Methods:**

Data were obtained from mortality registries covering 14 European countries. To determine educational inequalities in diabetes mortality, age-standardised mortality rates, mortality rate ratios, and slope and relative indices of inequality were calculated. To assess whether the association between education and diabetes mortality differs between genders, diabetes mortality was regressed on gender, educational rank and ‘gender × educational rank’.

**Results:**

An inverse association between education and diabetes mortality exists in both genders across Europe. Absolute educational inequalities are generally larger among men than women; relative inequalities are generally more pronounced among women, the relative index of inequality being 2.8 (95 % CI 2.0–3.9) in men versus 4.8 (95 % CI 3.2–7.2) in women. Gender inequalities in diabetes mortality are more marked in the highest than the lowest educated.

**Conclusions:**

Education and diabetes mortality are inversely related in Europe in the 2000s. This association differs by gender, indicating the need to take the socioeconomic and gender dimension into account when developing public health policies.

## Introduction

An individual’s disease risk cannot be separated from the population groups to which (s)he belongs (Elstad 2000). Important group-defining characteristics in this respect are socioeconomic position (SEP) and gender, with gender referring to the socially generated aspects of being a man/woman (Annandale 1998). Health and mortality differ systematically across SEP groups and gender. In contemporary western societies, the majority of health indicators are poorer among population groups with disadvantaged SEPs (Dalstra et al. 2008; Mackenbach et al. 2004). For gender, the picture is less univocal. Although women tend to be ill more often than men, they live longer. Despite biological factors playing a role, research has demonstrated that these differences are mainly socially generated (Case and Paxson 2005).

As for type 2 diabetes mellitus, there is an inverse gradient between SEP and both diabetes incidence and prevalence (Agardh et al. 2011; Espelt et al. 2008, 2013; Sacerdote et al. 2012), with some research reporting a rise in the magnitude of SEP inequalities in recent decades (Imkampe and Gulliford 2011; Smith 2007). The pattern is less clear for diabetes mortality, although most research does find an inverse association with SEP (Espelt et al. 2008; Forssas et al. 2003; Gnavi et al. 2004; Koskinen et al. 1996; Roper et al. 2001; Vandenheede et al. 2013). Furthermore, the majority of studies demonstrate a similar incidence and prevalence of type 2 diabetes mellitus in men and women (Gale and Gillespie 2001; Tang et al. 2003; Wild et al. 2004). This relative gender balance in incidence and prevalence does not hold for diabetes mortality. There is a clear preponderance of male diabetes deaths (Espelt et al. 2008; Romon et al. 2008).

Mounting evidence indicates that gender and SEP, as well as other social positions, do not operate in a vacuum, but often interact with each other (Macintyre and Hunt 1997; Sen et al. 2009). These interactive processes are not invariable, but vary within and between societies and over time (Macintyre and Hunt 1997), resulting in divergent implications for the design and development of public health policies (Sen et al. 2009). Although a burgeoning body of research shows that relative SEP inequalities in type 2 diabetes mellitus incidence, prevalence and mortality (Agardh et al. 2011; Dasgupta et al. 2010; Espelt et al. 2008, 2012, 2013; Robbins et al. 2001, 2005; Tang et al. 2003) are more pronounced in women relative to men, to the best of our knowledge, no study thus far has focused explicitly on the interaction between gender and SEP in relation to diabetes mortality. Assessing these associations may allow intricate insight into the mechanisms producing gender and SEP inequalities.

One way to gain insight into the mechanisms producing inequalities is cross-country research. To the extent that gender and SEP differences in diabetes mortality vary between countries, country characteristics are likely to be important determinants of these inequalities. Alternatively, finding no variation between countries would point to the universal nature of the phenomenon. The lion’s share of studies, assessing the relationship between SEP and/or gender and diabetes mortality, includes only one country (e.g., Gnavi et al. 2004; Landman et al. 2013; Romon et al. 2008; Vandenheede et al. 2013). So far we know, there is only one study that does compare SEP inequalities in diabetes mortality across Europe (Espelt et al. 2008). Using data from the 1990s and the beginning of the 2000s, Espelt et al. (2008) found an inverse educational gradient in diabetes mortality across Europe in men as well as in women, with gradients being particularly pronounced in women. The first objective of this study is to corroborate these results using more recent data from the 2000s on the one hand, and including countries and regions for which data were previously unavailable on the other (Austria, England and Wales, the Basque County, Madrid, Tuscany and Hungary). The second objective is to assess whether there is an interaction between gender, SEP and diabetes mortality. Probing into these associations may deepen our understanding of the mechanisms producing gender and SEP inequalities and, ultimately, open up new paths for diabetes prevention and care.

## Methods

### Design and study population

Table 1 describes the characteristics of the mortality studies included in the analyses. Data were gathered as part of the EURO-GBD-SE project. Both longitudinal (Denmark, Finland, Norway, Sweden, England and Wales, Austria, Belgium, Switzerland, the Basque Country, Madrid, Turin and Tuscany) and cross-sectional studies (Barcelona, Estonia, Czech Republic, Hungary and Poland) were used for this project. All longitudinal mortality datasets and the data from Barcelona consisted of linkage of census or population register data with the mortality register. In some countries (regions) (Austria, Madrid, Barcelona and the Basque Country, respectively), linkage was less than 95 % complete, and a correction factor was applied to the number of deaths in these regions to account for this. All studies covered the entire national territory, except for the Spanish and Italian datasets, and included all subgroups of the population, except for the Swiss mortality dataset, which is limited to Swiss nationals. The number of diabetes deaths was derived from the respective mortality registers, whereas person-years at risk was based on census or population register information. Person-years at risk was calculated using the number of subjects alive at the beginning of the study period times the length of the study period in the unlinked cross-sectional studies, and using the sum of the number of subjects alive at the beginning and end of each follow-up year divided by 2 in most longitudinal and repeated cross-sectional studies. The study population consisted of 30- to 74-year-olds. All mortality data refer to the 2000s.

**Table 1:** Main characteristics of the mortality studies for men and women aged 30–74 years (Europe, 2000s)

Country	Study design	Study period	Geographic coverage	Missing values on education	Men							Women					
					Person-years			Diabetes deaths				Person-years			Diabetes deaths		
					ISCED 0–2	ISCED 3–4	ISCED 5–6	ISCED 0–2	ISCED 3–4	ISCED 5–6		ISCED 0–2	ISCED 3–4	ISCED 5–6	ISCED 0–2	ISCED 3–4	ISCED 5–6
Nordic																	
Denmark	Longitudinal	2001–2005	National	–	2,520,048	3,054,351	1,636,288	1112	743	210		3,021,254	2,449,916	1,816,674	840	219	78
Finland^a^	Longitudinal	2001–2007	National	–	2,633,595	2,897,270	2,068,033	517	247	138		2,600,360	2,775,871	2,460,200	307	131	39
Norway	Longitudinal	2001–2006	National	1.8 %	1,037,652	3,294,516	1,492,898	330	376	70		1,214,131	3,129,174	1,477,289	235	147	26
Sweden	Longitudinal	2001–2006	National	1.1 %	3,906,490	7,304,043	2,792,904	1421	949	235		3607,241	6,895,713	3,582,960	921	465	103
United Kingdom																	
England and Wales^b^	Longitudinal	2001–2006	National	0.03 %	334,577	221,953	141,435	35	7	6		385,562	209,987	139,500	40	8	5
West, central																	
Austria	Longitudinal	2001–2002	National	–	450,564	1,416,447	385,445	112	170	12		902,055	1,201,818	232,099	150	55	6
Belgium	Longitudinal	2004–2005	National	2.4 %	2,615,875	1,437,699	1,461,630	466	88	65		2,826,164	1,400,978	1,478,930	421	26	22
Switzerland	Longitudinal	2001–2005	National	6.1 %	970,472	4,073,393	2,451,445	330	550	139		2,093,535	4,975,871	1,070,465	431	249	24
South																	
Barcelona	Repeated CS	2000–2006	City	–	1,632,272	821,816	819,752	334	38	56		1,995,574	730,001	859,413	208	16	12
Basque Country	Longitudinal	2001–2006	Region	–	1,658,243	801,562	653,416	246	23	33		1,944,112	590,384	665,050	203	6	0
Madrid	Longitudinal	2001–2003	Region	2.0 %	1,247,517	563,603	554,438	131	27	18		1,560,386	524,395	516,792	100	4	4
Turin	Longitudinal	2001–2006	City	–	635,631	345,160	170,043	153	27	13		779,568	331,206	161,131	106	9	5
Tuscany	Longitudinal	2001–2005	Florence, Leghorn, Prato	–	382,147	204,643	95,469	83	13	5		432,128	221,428	97,852	65	8	3
Baltic																	
Estonia	CS unlinked	1998–2002	National	2.2 %	473,750	890,770	281,075	81	96	23		492,655	1160,025	385,775	142	113	12
East, central																	
Czech Republic	CS unlinked	1999–2003	National	–	8,189,220	3,329,655	1,817,455	1333	222	49		8,560,640	4,406,360	1,234,640	1327	133	10
Hungary	CS unlinked	1999–2002	National	NA	3,886,828	5,107,684	1,497,752	1629	450	160		5,913,264	4,609,892	1,464,064	2206	221	49
Poland	CS unlinked	2001–2003	National	2.1 %	6,539,493	18,394,677	3,293,181	1744	2118	199		9,084,603	18,052,491	3,716,013	2987	1116	76

*CS* cross-sectional, *NA* not available

^a^The sample includes 80 % of the Finns

^b^Based on a 1 % sample

### Variables

Mortality from diabetes mellitus was defined by the International Classification of Diseases (ICD). Most studies used the 10th revision of the ICD (codes E10–14). However, definition of diabetes mortality was based on ICD-9 (code 250) in the Turin and Tuscan mortality data, and in the Austrian mortality data for the year 2001. All studies applied the standard epidemiological practice of only using the underlying cause of death.

The variable education was used as an indicator of SEP. Education was categorised according to the International Standard Classification of Education (ISCED), version 1997: pre-primary, primary and lower secondary education (ISCED 0–2); upper secondary and post-secondary non-tertiary education (ISCED 3–4); and tertiary education (ISCED 5–6). The proportion of missing values on education ranged from 0 % for most datasets to 6 % for Switzerland. Gender was included in the analyses as a dummy variable. The variable age was included as a categorical variable (5-year age bands) in the direct standardisation analyses, and as a continuous variable (mid-age of the 5-year age groups) in the Poisson regression analyses. Sensitivity checks with age as a categorical variable in both types of analyses demonstrated the robustness of the results. In some of the longitudinal datasets (Denmark, Norway, Sweden, Belgium and Switzerland), information on age was available at baseline only; while, for the other longitudinal and all cross-sectional datasets, age at the time of death was recorded. In datasets with age at baseline, people were not allowed to move into the next age group as they grew older. Hence, mortality estimates in these populations were higher compared to the estimates that would be obtained if age at death was used. To ensure comparability between mortality estimates, an adjustment procedure was developed that corrects for the upward bias in datasets which use age at baseline. Since only mortality rates are biased, the adjustment method was only applied when calculating mortality rates and absolute differences. Please refer to Östergren et al. (2001) for a detailed description of the adjustment formula.

### Data analysis

Cases with missing information on education were deleted from the analyses (complete-case analyses), and analyses were country and gender specific. To increase power, mortality data from Spain (Barcelona, the Basque Country and Madrid) and Italy (Turin and Tuscany) were grouped together.

First, to quantify the burden of diabetes mortality in each educational group, age-standardised mortality rates (ASMRs) and their 95 % confidence intervals (CIs) were computed, directly standardised to the 1976 European Standard Population.

Then, using age-adjusted Poisson regression models with person-years as the offset, both absolute and relative educational differences in diabetes mortality were estimated, allowing for a comprehensive picture of inequalities, with absolute inequalities being of more public health interest and relative inequalities being of more analytic interest. To measure absolute educational differences in diabetes mortality, the slope index of inequality (SII) was calculated. Relative educational differences were estimated through two different measures: the relative index of inequality (RII) and mortality rate ratios (MRRs). SII and RII represent the difference between the predicted diabetes mortality rates at the lower versus the higher end of the educational distribution. To estimate RII, a country- and gender-specific educational rank variable, ranging from 1 to 0 (lowest to highest end of the educational distribution), was calculated based on the ISCED-categorised education variable, and included in the Poisson models. SII was calculated using the following formula: ${{\left[ { 2\times {\text{ASMR}} \times \left( {{\text{RII}} - 1} \right)} \right]} \mathord{\left/ {\vphantom {{\left[ { 2\times {\text{ASMR}} \times \left( {{\text{RII}} - 1} \right)} \right]} {\left( {{\text{RII}} + 1} \right)}}} \right. \kern-0pt} {\left( {{\text{RII}} + 1} \right)}}$. As SII and RII account for differences in the distribution of education between countries and genders, they can be used for comparative purposes, but only on the condition of linearity between diabetes mortality rates and the educational rank variable (Wagstaff et al. 1991). Hence, in case of non-linearity, SIIs and RIIs are not presented. The MRRs represent the differences between the predicted diabetes mortality rates by education, using ISCED 5–6 as the reference group.

Next to these gender-specific analyses, age-adjusted Poisson regression models were fitted to test for interaction between gender, education and diabetes mortality. In addition to this, a pooled dataset, consisting of the data for all countries, was constructed to estimate the combined effect of education for all countries under study. Weights were assigned, so that the separate countries carried equal weight in the combined results.

## Results

Tables 2 and 3 present an overview of ASMRs per country by education for men and women, respectively. The general picture is one of the higher burden of diabetes mortality among men. With the exception of England and Wales and Estonia, ASMRs are higher in men than women. Among men, ASMRs (per 100,000 person-years) are between 10 and 15 in most countries; whereas, among women, ASMRs are generally between 5 and 10. All countries combined 14 diabetes deaths per 100,000 person-years occur in men, whereas the number of diabetes deaths in women is 9 per 100,000. The burden of diabetes differs also by education: diabetes mortality is higher among the lower educated in both men and women. Notably, the burden of diabetes mortality is particularly small among higher educated women, with the country-combined ASMR for higher educated women (ISCED levels 5–6) being 4.2 (95 % CI 2.7–5.8) per 100,000.

**Table 2:** Age-standardised diabetes mortality rates by education and country in men aged 30–74, and absolute and relative educational differences: slope index of inequality, mortality rate ratios and relative index of inequality (Europe, 2000s)

Country	Absolute inequalities					Relative inequalities			
	ASMR (95 % CI) per 100,000 person-years				SII	MRR (95 % CI)			RII (95 % CI)
	Overall	ISCED 0–2	ISCED 3–4	ISCED 5–6		ISCED 0–2^a^	ISCED 3–4^a^		
All countries	14.2 (13.2–15.3)	18.2 (16.1–20.3)	14.8 (13.0–16.2)	7.4 (5.8–9.1)	13.0	2.5 (1.9–3.1)	1.9 (1.6–2.6)		2.8 (2.0–3.9)
Denmark	24.0 (22.9–25.2)	32.2 (30.1–34.3)	21.9 (20.1–23.6)	12.7 (10.6–14.7)	24.0	2.4 (2.1–2.8)	1.7 (1.5–2.0)		3.0 (2.6–3.6)
Finland	11.0 (10.3–11.7)	15.1 (13.5–16.6)	10.0 (8.7–11.4)	7.2 (5.9–8.4)	10.4	2.0 (1.7–2.5)	1.4 (1.1–1.7)		2.8 (2.1–3.6)
Norway	11.2 (10.3–12.1)	18.1 (15.7–20.4)	10.7 (9.5–11.9)	4.4 (3.1–5.6)	14.4	3.6 (2.8–4.7)	2.3 (1.7–2.9)		4.6 (3.5–6.2)
Sweden	14.2 (13.6–14.8)	19.3 (18.1–20.6)	12.7 (11.7–13.6)	7.1 (6.0–8.2)	14.2	2.5 (2.2–2.8)	1.7 (1.5–2.0)		3.0 (2.5–3.4)
England and Wales	6.1 (4.3–7.8)	8.2 (5.3–11.0)	3.2 (0.8–5.6)	5.0 (1.0–9.0)	–	1.6 (0.7–3.9)	0.7 (0.2–1.9)		–
Austria	13.2 (11.7–14.8)	17.2 (13.8–20.6)	14.1 (11.9–16.3)	4.1 (1.8–6.4)	12.5	4.2 (2.3–7.7)	3.6 (2.0–6.5)		2.8 (1.8–4.4)
Belgium	9.3 (8.5–10.1)	11.6 (10.4–12.7)	7.2 (5.5–8.9)	5.0 (3.6–6.3)	10.7	2.5 (1.9–3.3)	1.7 (1.3–2.2)		3.7 (2.6–5.3)
Switzerland	10.8 (10.1–11.6)	17.7 (15.5–20.0)	11.2 (10.1–12.2)	5.2 (4.2–6.3)	13.1	3.0 (2.5–3.7)	2.0 (1.7–2.4)		4.1 (3.2–5.3)
Spanish regions	9.5 (8.9–10.2)	10.5 (9.7–11.3)	7.2 (5.6–8.7)	7.5 (6.1–8.9)	–	1.4 (1.1–1.7)	1.0 (0.7–1.3)		–
Italian regions	12.1 (10.6–13.5)	14.3 (12.4–16.3)	8.4 (5.8–11.0)	7.1 (3.8–10.4)	–	2.0 (1.2–3.2)	1.2 (0.7–2.1)		–
Estonia	12.0 (10.3–13.7)	14.6 (10.7–18.4)	14.0 (11.1–16.9)	8.5 (5.0–12.0)	–	1.5 (0.9–2.3)	1.6 (1.0–1.5)		–
Czech Republic	12.1 (11.5–12.7)	15.6 (14.8–16.5)	7.2 (6.3–8.2)	2.9 (2.1–3.8)	19.0	5.3 (4.0–7.1)	2.5 (1.8–3.4)		8.3 (6.3–10.8)
Hungary	20.7 (19.8–21.5)	27.2 (25.7–28.7)	15.1 (13.5–16.7)	10.4 (8.7–12.0)	23.8	2.5 (2.2–3.0)	1.4 (1.2–1.7)		3.7 (3.1–4.5)
Poland	15.0 (14.5–15.5)	18.0 (11.0–18.9)	16.0 (15.3–16.7)	6.6 (5.7–7.5)	9.3	2.6 (2.2–3.0)	2.4 (2.1–2.8)		1.9 (1.6–2.1)

RII and SII were not calculated, because of a non-linear relationship between diabetes mortality and the educational rank variable

*ASMR* age-standardised mortality rates, *CI* confidence interval, *MRR* mortality rate ratios, *RII* relative index of inequality, *SII* slope index of inequality

^a^The reference category was ISCED 5–6

**Table 3:** Age-standardised diabetes mortality rates by education and country in women aged 30–74, and absolute and relative educational differences: slope index of inequality, mortality rate ratios and relative index of inequality (Europe, 2000s)

Country	Absolute inequalities					Relative inequalities		
	ASMR (95 % CI) per 100,000 person-years				SII	MRR (95 % CI)		RII (95 % CI)
	Overall	ISCED 0–2	ISCED 3–4	ISCED 5–6		ISCED 0–2^a^	ISCED 3–4^a^	
All countries	8.9 (8.1–9.6)	12.2 (10.7–13.6)	6.9 (5.8–8.0)	4.2 (2.7–5.8)	12.1	3.1 (2.1–4.6)	1.8 (1.2–2.7)	4.8 (3.2–7.2)
Denmark	11.4 (10.6–12.2)	15.3 (14.0–16.5)	7.9 (6.7–9.1)	4.7 (3.4–6.0)	15.4	2.9 (2.3–3.7)	1.6 (1.2–2.0)	5.2 (3.9–7.0)
Finland	5.2 (4.7–5.7)	7.6 (6.4–8.8)	5.2 (4.3–6.2)	1.8 (1.2–2.4)	7.2	3.8 (2.7–5.4)	2.6 (1.8–3.7)	5.5 (3.6–6.3)
Norway	5.0 (4.4–5.6)	8.3 (6.9–9.8)	3.9 (3.2–4.6)	2.0 (1.0–3.0)	7.1	3.6 (2.4–5.4)	1.8 (1.2-2.7)	5.8 (3.7-9.0)
Sweden	7.0 (6.5–7.4)	10.8 (9.9–11.8)	5.6 (5.0–6.2)	2.8 (2.2–3.5)	9.5	3.7 (3.0–5.6)	2.1 (1.7–2.6)	5.2 (4.2–6.6)
England and Wales	5.9 (4.3–7.6)	6.6 (4.5–8.8)	4.7 (1.4–8.0)	3.6 (0.4–6.8)	–	1.6 (0.6–4.2)	1.0 (0.3–3.2)	–
Austria	7.8 (6.7–8.8)	9.3 (7.7–10.9)	5.5 (4.0–7.0)	6.1 (1.0–11.2)	7.6	1.8 (0.8–4.2)	1.1 (0.5–2.5)	2.9 (1.5–5.3)
Belgium	5.6 (5.1–6.2)	7.4 (6.6–8.3)	2.0 (1.1–3.0)	1.8 (0.8–2.7)	8.9	4.0 (2.6–6.2)	1.8 (1.2–2.9)	8.6 (5.3–13.9)
Switzerland	5.8 (5.3–6.3)	8.5 (7.5–9.6)	4.2 (3.6–4.8)	2.2 (0.9–3.4)	7.3	3.0 (1.9–4.5)	1.5 (1.0–2.3)	4.4 (3.2–6.0)
Spanish regions	4.6 (4.2–5.0)	5.1 (4.6–5.6)	2.9 (1.8–4.1)	1.9 (0.9–2.8)	–	2.8 (1.7–4.5)	1.6 (0.9–3.0)	–
Italian regions	6.4 (5.5–7.4)	7.1 (6.0–8.3)	3.7 (1.9–5.4)	4.1 (1.2–7.0)	–	1.7 (0.8–3.5)	0.9 (0.4–2.2)	–
Estonia	10.7 (9.4–12.0)	16.0 (11.4–20.6)	10.8 (8.8–12.7)	3.4 (1.5–5.4)	–	4.1 (2.3–7.5)	3.2 (1.8–5.8)	–
Czech Republic	8.7 (8.2–9.1)	10.3 (9.5–10.9)	4.5 (3.7–5.3)	1.4 (0.5–2.3)	13.7	8.0 (4.3–14.9)	3.4 (1.8–6.4)	8.5 (5.9–12.2)
Hungary	16.1 (15.5–16.8)	19.8 (18.9–20.7)	7.7 (6.6–8.7)	5.8 (4.1–7.5)	25.0	3.8 (2.9–5.1)	1.5 (1.1–2.0)	8.0 (6.2–10.5)
Poland	11.6 (11.2–11.9)	15.2 (14.5–15.9)	8.9 (8.4–9.4)	3.4 (2.6–4.2)	13.9	4.7 (3.7–5.9)	2.8 (2.2–3.5)	4.0 (3.5–4.6)

RII and SII were not calculated, because of a non-linear relationship between diabetes mortality and the educational rank variable

*ASMR* age-standardised mortality rates, *CI* confidence interval, *MRR* mortality rate ratios, *RII* relative index of inequality, *SII* slope index of inequality

^a^The reference category was ISCED 5–6

There is an inverse association between education and diabetes mortality in all countries in both genders. In most countries, this association takes on the form of a gradient, as is the case in, for example, Danish men and Belgian women. In Danish men, ASMRs are 32.2 (95 % CI 30.1–34.3), 21.9 (95 % CI 20.1–23.6) and 12.7 (95 % CI 10.6–14.7) among the lowest (ISCED 0–2), mid- (ISCED 3–4), and highest educated (ISCED 5–6), respectively (Table 2). MRRs among Belgian women are 4.0 (95 % CI 2.6–6.2) for the lowest educated and 1.8 (95 % CI 1.2–2.9) for the mid-educated, relative to the highest educated women (Table 3). In other countries, such as England and Wales, there is an inverse association between education and diabetes mortality, where the ISCED 0–2 group has the highest mortality, but the relationship is not graded.

### The interaction between gender and education

With the exception of Hungary and Poland, absolute educational inequalities are generally larger in men compared to women. For example, the SII among Norwegian men amounts to 14.4 per 100,000 person-years, while the SII among Norwegian women is 7.1 per 100,000 (Tables 2, 3). Overall, the SII is 13.0 in men and 12.1 in women. Absolute inequalities are slightly higher in men relative to women. On the other hand, relative educational inequalities are generally larger in women. For example, the RII among Hungarian women is 8.0 (95 % CI 4.6–16.1) versus 3.7 (95 % CI 3.1–4.5) among Hungarian men. For all countries combined, the estimated mortality risk in the lowest educated men is almost 3 times as high as in the highest educated men; while for women, this figure is nearly 5.

Table 4 focuses specifically on gender differences in educational inequalities in diabetes mortality. It presents the results from the age-adjusted Poisson regression models, which test for the interaction between gender, educational rank and diabetes mortality. Table 4 shows, on the one hand, that relative educational inequalities are more marked among women than men; on the other, that gender inequalities in diabetes mortality are more pronounced among the higher educated than the lower educated. This pattern is remarkably similar across countries. All countries combined, the burden of diabetes mortality in the highest educated women is 0.4 times that of the highest educated men, while the burden of diabetes mortality in the lowest educated women is 0.8 times (0.4 × 2.1) the burden in the lowest educated men.

**Table 4:** Age-adjusted exponentiated diabetes mortality coefficients for the educational rank variable, gender and the product term educational rank × gender in men and women aged 30–74 (Europe, 2000s)

Country	Exponentiated coefficients (95 % CI)		
	Educational rank^a^	Gender^b^	Educational rank × gender
All countries^c^	2.6 (2.2–3.3)	0.4 (0.3–0.5)	2.1 (1.6–2.4)
Denmark	3.0 (2.6–3.6)	0.3 (0.3–0.4)	1.8 (1.3–2.5)
Finland	2.7 (2.1–3.6)	0.3 (0.2–0.4)	2.0 (1.3–3.3)
Norway	4.4 (3.3–5.8)	0.3 (0.2–0.5)	1.6 (0.9–2.6)
Sweden	3.0 (2.6–3.5)	0.4 (0.3–0.4)	1.7 (1.3–2.2)
Austria	2.6 (1.7–4.0)	0.5 (0.3–0.8)	1.2 (0.6–2.6)
Belgium	3.1 (2.2–4.5)	0.2 (0.1–0.4)	4.1 (2.0–8.5)
Switzerland	3.7 (2.9–4.7)	0.4 (0.3–0.5)	1.4 (1.0–2.2)
Czech Republic	8.2 (6.3–10.7)	1.1 (0.7–1.8)	0.6 (0.5–0.8)
Hungary	3.4 (2.8–4.1)	0.4 (0.3–0.5)	2.8 (2.0–3.8)
Poland	1.6 (1.5–1.8)	0.4 (0.3–0.4)	3.1 (2.6–3.7)

*CI* confidence interval

^a^The educational rank variable ranged from 1 (lowest end of the educational distribution) to 0 (highest end of the educational distribution)

^b^The reference category was men

^c^All countries combined, the relationship between the educational rank variable and diabetes mortality was linear. Thus, for the calculation of the “all countries” estimates, England and Wales, the Spanish regions, the Italian regions, and Estonia were included as well

## Discussion

### Summary of findings

This study indicates that education is inversely related to mortality from diabetes mellitus across Europe in both men and women. Absolute educational inequalities are generally larger in men than in women, reflecting men’s higher diabetes mortality. Relative educational inequalities in diabetes mortality are more marked among women than men. There is an interaction effect between education, gender and diabetes mortality in most European countries, with the burden of diabetes mortality being particularly small among the highest educated women. All countries combined, diabetes mortality among the highest educated women is 0.4 times that of the highest educated men, while diabetes mortality among the lowest educated women is 0.8 times that of the lowest educated men.

### Methodological considerations

Data were derived from both longitudinal and cross-sectional mortality studies. All longitudinal data and data from the repeated cross-sectional study were linked. Yet, all datasets, from Eastern European and Baltic countries, used an unlinked study design. Unlinked data are more prone to numerator–denominator bias, since misclassification between the numerator (number of deaths, derived from mortality register) and the denominator (number of person-years, derived from census/population register) of the rates might occur. However, as educational and gender patterns in diabetes mortality do not differ markedly between linked and unlinked studies, the effect of numerator–denominator bias on the magnitude of inequalities is expected to be rather small.

Another methodological consideration relates to the analysis of cause-specific mortality data at a European level. Comparing cause-specific mortality between European countries inevitably raises questions concerning the comparability of cause-specific death registration. There may be incongruities between European countries in death certificate models; nature and amount of information entered; application of the rules for selection of underlying, intermediary and associated causes of death and other coding practices; ICD-revision used; implementation of automated coding systems (Meslé 2002). These discrepancies make it difficult to compare the absolute burden of cause-specific mortality between countries. When it comes to diabetes, some discrepancies may be rather minor. For example, a bridge-coding study, calculating cause-specific mortality estimates using both ICD-9 and ICD-10 indicated an excellent comparability for diabetes. There was a less than 1 % increase in diabetes deaths using ICD-10 instead of ICD-9 (Anderson et al. 2001). Additional cross-country comparison difficulties are related to ambiguities in the role of diabetes as a cause of death. Since diabetes is often considered part of a complex clinical picture, it is mostly registered as one of the contributing causes, not as the underlying cause of death. To the extent that the use of diabetes as an underlying/contributing cause differs between countries, cross-country comparisons of diabetes mortality are difficult to interpret (Jougla et al. 1992).

As in most other studies on diabetes mortality (Espelt et al. 2008; Gnavi et al. 2004; Roper et al. 2001), we were unable to distinguish between mortality from type 1 and mortality from type 2 diabetes mellitus. The bias introduced by this merging together is considered to be minimal, since approximately 90 % of all people with diabetes do have type 2 diabetes (Dawson 2009). The frequent registration of diabetes mellitus as contributing instead of underlying cause leads to a severe underestimation of the burden of diabetes mortality—by 150–400 % depending on the study population—because of the standard epidemiological practice of using the underlying cause of death only (Romon et al. 2008; Vandenheede et al. 2011).

On the condition that registration practices are the same across educational groups and genders within countries, relative differences are not affected. Therefore, we decided not to emphasise the differences between countries in ASMRs or SIIs, but instead to focus on the general pattern and on the differences between countries in RIIs. Relative differences in diabetes mortality as found in our analysis echo findings from incidence and prevalence data (Agardh et al. 2011; Espelt et al. 2008, 2013; Sacerdote et al. 2012). The inverse graded association between education and diabetes mortality reflects the educational pattern of diabetes incidence and prevalence (Agardh et al. 2011; Espelt et al. 2008, 2013; Sacerdote et al. 2012), indicating that differential diabetes mortality by education is not solely due to differential registration practices, but mainly reflects actual differences in diabetes mortality.

Several advantages and disadvantages are associated with the use of education as a measure of SEP. Main advantages are: (1) its universality, it is applicable to people regardless of age and working circumstances; (2) its comparability; (3) the relative ease with which it can be measured; and (4) its relative insensitivity to health-related selection, and thus to reverse causality (Galobardes et al. 2007). Despite its general applicability, education lacks a universal meaning, since its implications are related to gender, ethnicity, birth cohort, and social class, amongst other things. Other disadvantages are mainly associated with the fact that education captures only a part of SEP, i.e. knowledge-related skills and assets rather than material circumstances (Galobardes et al. 2007). Yet, as both education and diabetes mortality are strongly lifestyle related, education may be a particularly relevant indicator for studying SEP inequalities in diabetes mortality. Furthermore, despite being attained rather early in life, education has been shown to remain as a key indicator of SEP through the life course.

### Theoretical considerations

#### Educational inequalities in diabetes mortality

In line with the most previous studies (Espelt et al. 2008; Tang et al. 2003; Vandenheede et al. 2013), a strong inverse association between education and diabetes mortality was found among both men and women. Our results are similar to the ones of Espelt et al. (2008), indicating that the educational patterning of diabetes mortality found in the 1990s largely persists in the 2000s. If there is any change between the two periods, it is an increase in the magnitude of the association among men. Other studies also report a rise in the magnitude of SEP inequalities in diabetes in recent decades (Imkampe and Gulliford 2011; Smith 2007). We also observed an inverse association between education and diabetes mortality in countries and regions for which data were previously unavailable.

Several explanations for SEP inequalities in diabetes mortality have been put forward. Analogous to explanations for educational differences in diabetes incidence and prevalence, lifestyle, and more specifically obesity, is considered to be a key intermediary factor (Espelt et al. 2013; Vandenheede et al. 2013). Furthermore, factors related to diabetes progression, such as access to and quality of diabetes care, metabolic control and complications, could also be important determinants (Bachmann et al. 2003). Since diabetes management is becoming ever more technical and demanding, and since higher educated groups are better equipped to take full advantage of these developments than lower educated groups, educational inequalities in diabetes management and mortality are likely to widen (Phelan et al. 2010; Smith 2007). Another explanation for the rather strong association of education with diabetes mortality could be related to attitudes towards health. While higher-SEP people are inclined to think about their future health risk, to consider themselves as having control over their health, and to be aware of the influence of lifestyle on health, lower-SEP people are less prone to do so (Wardle and Steptoe 2003). These SEP differences in lifestyle, diabetes progression factors and attitudes cannot be separated from the differences in life opportunities and material circumstances between SEP groups, which may themselves arise because of the macro socioeconomic and political context (CSDH 2010).

The enduring and consistent relationship between education and diabetes mortality in most European countries, despite huge variation in macro-economic and political characteristics, points to the persistent nature of health inequalities (Phelan et al. 2010). Furthermore, it suggests that it is difficult to breach these inequality patterns and develop policies that give equal chances to all. Despite educational patterns in diabetes mortality being similar across Europe, inequalities are of different magnitudes in different countries. Hence, it would be of importance for future research to include country variables in order to determine how country characteristics affect educational inequalities in diabetes mortality. Doing so, we can gain further insight into inequality-generating mechanisms and fine-tune policies to reduce these inequalities.

#### A consistent gender pattern

We observed a higher burden of diabetes mortality among men relative to women, supporting previous evidence (Espelt et al. 2008). A possible explanation for this observation is differences in diabetes progression, as diabetes incidence and prevalence are highly comparable between men and women (Gale and Gillespie 2001; Wild et al. 2004). Previous studies have demonstrated a differential pattern of complications between men and women (Abbate et al. 2012), differences in diabetes management and in use of health care (Krämer et al. 2012). Another possible explanation is that many deaths from diabetes involve cardiovascular complications. Since the background risk of mortality from cardiovascular disease is higher among men than women, mortality risks of people with diabetes may be higher among men than women.

A larger SEP gradient in diabetes incidence (Espelt et al. 2013) prevalence (Espelt et al. 2008, 2013) and mortality (Espelt et al. 2008) among women relative to men has been established in previous research. However, to our knowledge, none of these studies has examined the interaction between gender, SEP and diabetes mortality explicitly. The interaction can be examined from different perspectives: relative educational differences are larger among women than men, and gender differences are more pronounced among the highest versus the lowest educated. The larger relative inequalities among women compared to men may be related to the smaller overall diabetes mortality levels among women, since there is an empirical relationship between relative mortality inequalities and the overall mortality level. The lower the burden of mortality is, the higher relative differences generally are (Houweling et al. 2007). Furthermore, larger educational differences in diabetes mortality among women mirror larger educational inequalities in some highly prevalent chronic diseases such as cardiovascular disease (Dalstra et al. 2008), and have been explained by the more pronounced gradients in obesity and related lifestyle factors among women relative to men (Espelt et al. 2013). Among higher-SEP women, prevalence of obesity, one of the major risk factors of diabetes, is rather low. One of the main explanations given in the literature is that higher-SEP women apply stricter behavioural norms and thinness ideals than both men and lower-SEP women (Roskam et al. 2010). The very small burden of diabetes mortality among the highest educated women mirrors the obesity findings. Hence, the explanation may be rather similar as well. Highly educated women are a very health-conscious group. They are more committed to their health than both lower educated women and men, and engage more in preventive efforts (Annandale 1998). The very small burden of diabetes mortality among this group of women ultimately shows that diabetes mortality is highly preventable, and, hence, an unnecessary cause of death.

### Conclusions and recommendations

There is a need for studies probing into the mechanisms behind the interaction between gender, education and diabetes mortality, and for research into the persistence of health inequalities over time and in different settings. The low burden of diabetes mortality among higher educated women suggests large possibilities for intervention (e.g., by prevention and treatment of obesity). The relatively high burden of diabetes mortality among higher educated as well as lower educated men indicates that there are multiple barriers in men to effectively engage in diabetes management and care. Future policies should aim at reducing these barriers, while simultaneously improving diabetes care for all. One possible track for both the improvement of diabetes care and the reduction of inequalities in diabetes care may be close cooperation between general practitioners and endocrinologists (“shared care”) (Gnavi et al. 2009).
